# Efficacy of topical and systemic transplantation of mesenchymal stem cells in a rat model of diabetic ischemic wounds

**DOI:** 10.1186/s13287-021-02288-8

**Published:** 2021-03-31

**Authors:** Jianxin Yan, Jiaji Liang, Yingxuan Cao, Mariya M. El Akkawi, Xuan Liao, Xiaojia Chen, Chengzhi Li, Kecheng Li, Guanghui Xie, Hongwei Liu

**Affiliations:** 1 Department of Plastic Surgery, The First Affiliated Hospital of Jinan University, Guangzhou, 510630 People’s Republic of China; 2 Innovative Technology Research Institute of Plastic Surgery, Guangzhou, 510630 People’s Republic of China; 3 Key Laboratory of Regenerative Medicine, Ministry of Education, Guangzhou, 510632 People’s Republic of China; 4 Department of Cell Biology & Institute of Biomedicine, College of Life Science and Technology, Jinan University, Guangzhou, 510632 People’s Republic of China; 5 Guangdong Province Key Laboratory of Bioengineering Medicine, Guangzhou, 510632 People’s Republic of China; 6 Guangdong Provincial Biotechnology Drug & Engineering Technology Research Center, Guangzhou, 510632 People’s Republic of China; 7 National Engineering Research Center of Genetic Medicine, Guangzhou, 510632 People’s Republic of China; 8 Department of Interventional Radiology and Vascular Surgery, The First Affiliated Hospital of Jinan University, Guangzhou, 510630 People’s Republic of China

**Keywords:** Bone marrow-derived mesenchymal stem cells, Type 2 diabetes mellitus, Critical limb ischemia, Diabetic foot ulcers, Chronic wounds

## Abstract

**Background:**

Mesenchymal stem cells (MSCs) exert positive effects in chronic wounds. However, critical parameters, such as the most effective administration routes, remain unclear. Accordingly, the purpose of this study was to compare the effects of topical and systemic transplantation MSCs on diabetic ischemic wound healing and explored the underlying mechanisms.

**Method:**

A diabetic ischemic wound model was created on the dorsal foot of type 2 diabetes mellitus (T2DM) rat. Bone marrow-derived mesenchymal stem cells (BM-MSCs) were administered via two routes: topical injection and intravenous (IV) infusion. Wound healing outcomes and blood glucose level were assessed dynamically. Meanwhile, blood flow recovery was evaluated in ischemic gastrocnemius muscles. The homing and transdifferentiation of mKate2-labeled BM-MSCs were assessed by fluorescence imaging and immunohistochemistry (IHC) analysis.

**Result:**

Both topical and systemic treatments had a positive effect on the diabetic ischemic wound showing a significant reduction in wound area at day 14. Histological results showed an increase in the length of epithelial edges, collagen content, microvessel density in the wound bed, and a higher expression of vascular endothelial growth factor (VEGF). Meanwhile, systemic administration can ameliorate hyperglycemia and improve the blood perfusion of the ischemic hindlimb. BM-MSCs administered systemically were found distributed in wounded tissue and transdifferentiated into endothelial cells. Furthermore, BM-MSCs stimulated angiogenesis at wound sites by downregulating phosphatase and tensin homolog (PTEN) and activation of AKT signaling pathway.

**Conclusions:**

The results demonstrated that both transplantation delivery method (topical and systemic) of BM-MSCs accelerated wound healing remarkably under pathological conditions. Nevertheless, systemic administration has the potential to ameliorate hyperglycemia and repair the damaged tissue.

## Introduction

Type 2 diabetes mellitus (T2DM) is the most prevalent form of diabetes, accounting for 90 to 95% of existing cases. T2DM is characterized by insulin resistance in the target tissue, a relative lack of insulin secretion, and subsequent decline in the β cell function in the pancreas [1]. In clinical practice, patients suffering from T2DM frequently present with critical limb ischemia and foot ulcer [2]. Diabetic foot ulcers (DFUs) are one of the most common types of chronic wounds with pathological hallmarks of decreased vascularization, elevated oxidative stress, and infection [3]. The occurrence of DFUs is closely related to peripheral arterial disease (PAD), which is a process of chronic atherosclerosis, mainly resulting in the narrowing of the lower limbs’ peripheral arterial vascular system. Clinical data suggested that DFUs is one of the major causes of non-traumatic lower limb amputation, mainly due to microvascular and macrovascular complications [4]. At present, the management of DFUs includes glycemic control, pharmacological therapy, improving vascularization, debridement, “offloading” strategies, wound dressings, negative pressure wound therapy, maggot therapy, growth factors, and skin substitutes [5]. However, these conventional treatments have certain limitations.

In recent years, studies showed the effectiveness of mesenchymal stem cells (MSCs) in treating DFUs [6, 7]. As one of multipotent adult stem cells, MSCs can differentiate into the different cell lineages in damaged tissues by activating endogenous progenitor cells and secreting various factors and regulating their local environment [8, 9]. Therefore, MSCs have great application prospects in the regenerative treatment of damaged tissues and chronic wounds, such as critical limb ischemia [10], diabetic foot [11], and pressure ulcers [12]. Although a spectacular number of MSCs studies have already been conducted, the safety and efficacy of MSCs still remains uncertain: in terms of source, management prior to administration, nature (autologous or allogenic), optimum dosage, and route and timing of administration. Systemic administration and topical delivery are both important methods for MSC transplantation. Intravenous (IV) infusion is the common method of clinical systemic administration due to being a relatively easy and least invasive procedure [13]. However, it was reported that MSCs follow a process known as “lung entrapment” which leads to cells mainly distributed in the lungs and rarely homing to the target tissue after IV infusion [14, 15]. Lung entrapment is also considered to be a first-pass effect in the early stages (1 h) after IV infusion and MSCs homing in other tissues and organs over time [16]. Whether MSCs can reach target tissues through IV infusion remains controversial. Hence, recent research on MSCs therapy of wounds focused on topical transplantation, which directly injects MSCs into the target tissue. Regardless, both systemically and locally administered MSCs promote wound healing under physiological conditions, but their mechanism may be different [17]. As a result, the optimal route of transplantation of MSCs for chronic wounds requires further research.

In this study, a diabetic ischemic wound model on rat dorsal foot was designed to mimic the pathophysiology of clinical patients who suffer from DFUs. Bone marrow-derived MSCs (BM-MSCs) are currently the most frequently used MSCs source, so we transplanted BM-MSCs topically and systemically to investigate the proper administration method for chronic wound therapy. The potential therapeutic mechanisms were further investigated.

## Materials and methods

### Isolation, culture, identification, and labeling of BM-MSCs

BM-MSCs were collected by flushing the femurs and tibias of 4-week-old male Sprague-Dawley rat (Guangdong Medical Laboratory Animal Center). These cells were cultured at 37 °C with 5% CO_2_ in a basic medium, consisting of high glucose Dulbecco’s modified Eagle’s medium (DMEM; Product # 10–013-CVR; Corning), 10% fetal bovine serum (FBS; Product # 04–001-1ACS; Biological Industries), and 1% penicillin-streptomycin (pen/strep; Product # SV30010; HyClone). Nonadherent cells were removed after 48 h of incubation and a fresh medium was added. The medium was changed every 48 or 72 h and further propagated the adherent spindle-shaped cells for three passages.

BM-MSCs were identified by immune phenotypic analyses and differentiation studies as described previously [18]. We phenotypically characterized the cultured cells for the expression of MSC markers by flow cytometry as CD29^+^ CD44^+^ CD90^+^ CD34^−^ CD45^−^. On the other hand, differentiation into adipocytes, osteocytes, and chondrocytes determined their functional characterization. Three days after transfecting mKate2-expressing-lentivirus to BM-MSCs (passage 2) at 70% cell confluence, fluorescence microscope (IX51; Olympus) was used for the detection of infection efficiency, and the cells were cultured successively.

### Diabetic ischemic wound model

Seven-week-old 200 g male Sprague-Dawley rats (Guangdong Medical Laboratory Animal Center) were used for all studies, maintained on a 12-h:12-h light-dark cycle under ad libitum feeding and water consumption. The animal experiment protocol was approved by the Laboratory Animal Ethics Committee of Jinan University. The T2DM model was induced in rats with fat-fed (45% fat/35% carbohydrate/20% protein; and 4.73 kcal/g) 2 weeks and then streptozotocin injected intraperitoneally twice (24 h apart) at a dose of 35 mg/kg [19, 20]. Intraperitoneal glucose tolerance tests (IPGTTs), intraperitoneal insulin tolerance tests (IPITTs), and serum insulin were performed 1 week after STZ injection to confirm the T2DM rat model. Blood glucose monitor (Roche Diagnostics GmbH, Mannheim, Germany) was used to measure blood glucose. Quantitative measurements of serum insulin were performed by ELISA (Rat Insulin Elisa Kit; Wuxi Donglin Sci & Tech Development Co., Ltd., Jiangsu, China).

Twelve weeks following the success of T2DM model, serum nitric oxide (NO) (Nitric Oxide assay kit; NanJing JianCheng Bioengineering Institute Co., Ltd., Nanjing, China), serum endothelin-1 (ET-1) (Rat Endothelin-1 ELISA Kit; Wuhan Huamei Bioengineering Co., Ltd., Wuhan, China), and oil red O staining (Wuhan servicebio technology Co., Ltd., Wuhan, China) were performed to confirm the vasculopathy. Meanwhile, body weight, 24-h water, and feed intake were compared between normal and T2DM rats. After that, a single round full-thickness skin wound was created using disposable 5 mm skin biopsy punch in T2DM rats after the ligation of the right femoral artery to created a diabetic ischemic wound model on the dorsal hindfoot.

### Transplantation and tracing of BM-MSCs

Rats with diabetic ischemic wounds were randomly divided into a PBS control group, a BM-MSC topically treated group, and a BM-MSC IV-treated group. Topically treated rats received 1 × 10^6^ BM-MSCs in 100 μL of PBS intradermal injections at four injection sites around the wound immediately after creation [21]. In contrast, IV treated rats received 5 × 10^6^ BM-MSCs in 1000 μL PBS via the left femoral vein right immediately after wound creation [6]. To evaluate the distribution of MSCs in this model at days 3, 7, and 14, the mKate2-labeled BM-MSCs were applied in the same dosage and administration method as previously described to trace using fluorescence imaging and immunohistochemistry (IHC) analysis.

Rats were perfused with 60 ml of normal saline through the left ventricle followed by 100 ml of 4% paraformaldehyde. After perfusion, the major organs (Fig. 6a) were dissected and subjected to fluorescence imaging. Fluorescence imaging was performed using In Vivo Xtreme Imaging System (Bruker Corporation, Karlsruhe, Germany) with excitation wavelength 588 nm and emission wavelength 635 nm. For IHC analysis, the lung, pancreas, and wound sites were detected with antibodies specific against mKate2 (1:4000; Product # R10367; Invitrogen), the latter subsequently visualized with Alexa Fluor® 647 Goat Anti-Rabbit IgG antibody (1:500; Product # A-21245; Invitrogen).

### Measurement of wound contraction rate and blood glucose level

Digital photographs of wounds were taken and blood glucose level was determined at days 0, 3, 7, 10, and 14. The wound area was measured using ImageJ analysis software (National Institutes of Health, Bethesda, MD) by tracing the wound margin. The wound area healing rate was calculated as follows:

Wound area healing rate (%) = ([area of original wound - area of actual wound]/[area of original wound]) × 100.

### Color-coded quantitative digital subtraction angiography (q-DSA)

At days 0, 7, and 14 after injury, double hindlimb q-DSA was performed by abdominal aorta injection of 4 mL Iohexol (320 mgI/ml) at 1 mL/s speed, 111 kPa pressure. DSA scans were acquired at a frame rate of 6 frames per second (F/s) with the Siemens Artis Zeego C-arm, combined with Artis Zeego software from Siemens Healthineers (version VD11D, Siemens Healthcare, Erlangen, Germany). Image analysis took place using syngo iFlow postprocessing software (Siemens Healthineers, Forchheim, Germany). The gastrocnemius regions of the hindlimb on both sides are selected symmetrically as the region of interest (ROI), and the right ROI peak (PeakROI-R) and left ROI peak (PeakROI-L) are obtained. Then, the ratio of the PeakROI-R to the PeakROI-L was calculated (rPeak = PeakROI-R/PeakROI-L).

### Histological assessment

The wound (including 2 mm of the surrounding skin) and the right gastrocnemius tissue samples were harvested at days 3, 7, 10, and 14. The sections of the wound tissue were stained with hematoxylin and eosin (H&E) and with Masson’s trichrome following the manufacturers’ protocol (Wuhan servicebio technology Co., Ltd., Wuhan, China) to detect the reepithelialization/granulation tissue formation and collagen deposition, respectively. Meanwhile, the anti-CD31 antibody (1:300; Product # ab182981; ABcam) and the anti-VEGF antibody (1:200; Product # ABS82; Sigma-Aldrich) IHC were performed. Goat anti-rabbit IgG H&L (HRP) (1:200; Product # ab205718; ABcam) was used as a secondary antibody. The length of both the epithelial edges and the epithelial gap, as well as the area of the granulation tissue on the wound, were determined (Fig. 4a) according to the criteria described previously [22]. Microvessel density is expressed as the number of stained vessels per high power field (HPF) (Fig. 5) refer to previous study [23]. The positive areas of collagen, mKate2, and VEGF staining were calculated using ImageJ analysis software as follows:

Collagen content/area of mKate2 staining/VEGF content (%) = ([area of positive staining]/[area of the entire field of view]) × 100.

### Double-labeling IHC for wounded tissue

After administration on day 14, wound and right gastrocnemius sections were incubated with anti-mKate2 rabbit polyclonal antibody (1:4000; Product # R10367; Invitrogen) and anti-CD31 mouse monoclonal antibody (1:1500; Product # ab24590; ABcam) overnight at + 4 °C, followed by a further incubation at room temperature for 1 h with Alexa Fluor® 647 goat anti-rabbit IgG antibody (1:500; Product # A-21245; Invitrogen) and Alexa Fluor® 488 goat anti-mouse IgG antibody (1:500; Product # ab150117; ABcam). Nuclei were counterstained with DAPI stain (Product # G1012; Servicebio).

### Western blot analysis

Total protein was extracted from samples of wound by Total Protein Extraction Kit (Beyotime Institute of Biotechnology, Shanghai, China) at day 14 post-treatment.

Equal amounts of total protein were separated on 10% SDS-PAGE and transferred to nitrocellulose membranes. Membranes were incubated overnight at 4 °C with monoclonal antibody against VEGF (Product # ABS82), phosphorylation of AKT (p-AKT) (Product # SAB4504331), AKT (Product # SAB4500797), phosphatase and tensin homolog (PTEN) (Product # SAB4300337), and β-actin (Product # ABT264) (all 1:1000; Sigma-Aldrich). Then, the membranes were incubated with HRP-conjugated anti-rabbit (1:5000; Product # ab205718; ABcam).

### Statistical analysis

All data were presented as means ± SD. Two analytic methods, *t* test and ANOVA, were used to analyze the experimental data. Statistically significant was represented as *P < 0.05, **P < 0.01, and ***P < 0.001.

## Results

### Identification of BM-MSC characteristics

**Fig. 1:**
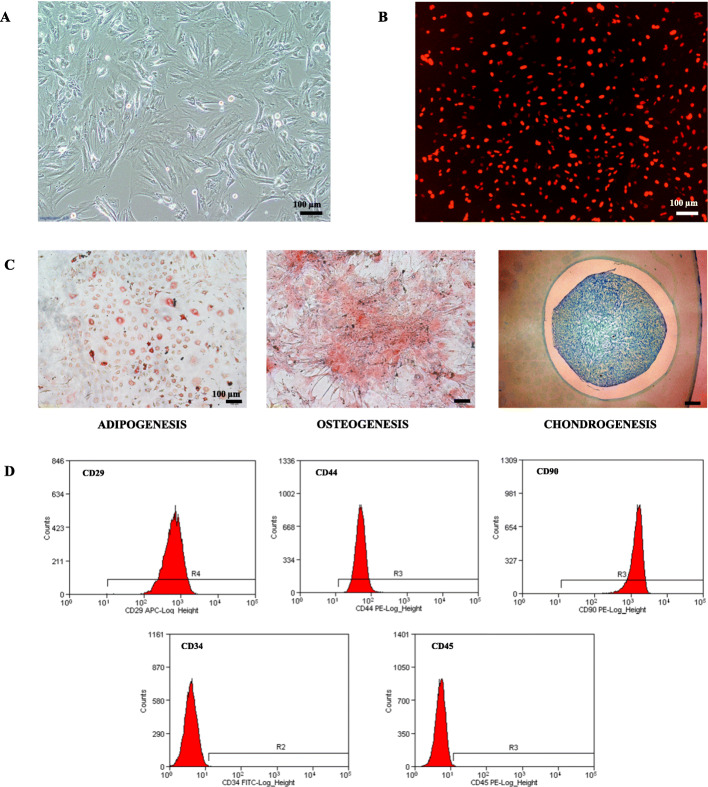
Characterization of rat BM-MSCs. **a** Brightfield image of BM-MSCs in culture. **b** A fluorescent image of transduced BM-MSCs expressing mKate2 (red fluorescence). **c** Adipogenic differentiation was revealed with oil red O staining. Osteogenic differentiation was confirmed by alizarin red staining. The chondrogenic potential of MSCs was determined by staining for Alcian blue. **d** Cell surface markers of MSCs were assessed using flow cytometry. MSCs expressed CD29, CD44, and CD90, but not CD34, CD45

Isolated cells were plastic-adherent in culture and displayed a typical fibroblast morphology as shown in Fig. 1a. mKate2 fluorescence was visible in the nucleus of the transduced cells demonstrated in Fig. 1b. Also, cultured cells were positive for oil red O staining, alizarin red staining, and Alcian blue staining (Fig. 1c). As shown in Fig. 1d, 95% of these cells were positive for CD29, CD44, and CD90, but negative for CD34 and CD45 indicating that the cultured cells possessed the MSC characteristics [24].

### Characteristics of the T2DM and vasculopathy

**Fig. 2:**
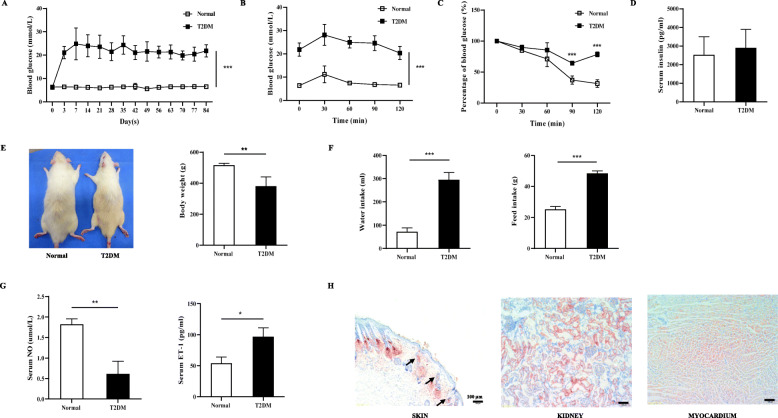
Characterization of T2DM and vasculopathy in rats. **a** Time course of random blood glucose levels following STZ. One week after STZ injection, glucose tolerance was assessed by IPGTTs, by intraperitoneal injection of 2 g glucose/kg body weight and determining blood glucose levels (**b**); insulin tolerance was evaluated by IPITTs, by injecting 2 g glucose/kg body weight immediately followed by insulin administration at a dose of 2 units/kg body weight (**c**); and serum insulin levels were evaluated by ELISA (**d**). 12 weeks after STZ injection, representative picture, and body weight (**e**); 24-h water intake and 24-h feed intake (**f**); Serum NO and serum ET-1 levels were evaluated by ELISA (**g**); Skin vessels (black arrow), kidney, and myocardium lipid accumulation in T2DM rats (**h**). Values are mean ± SD. *P < 0.05, **P < 0.01, ***P < 0.001

Blood glucose, IPGTTs, IPITTs, and serum insulin were measured to confirm the success of T2DM rat model. One week after STZ injection, blood glucose levels in the T2DM group increased to more than double that of normal rats (*p* < 0.001, Fig. 2a). The results of IPGTTs revealed significant deterioration in glucose metabolism (Fig. 2b), and IPITTs showed a significant decrease in insulin sensitivity (Fig. 2c). However, there was no significant difference in serum insulin levels between the normal and T2DM rats (Fig. 2d). After 12 weeks of STZ injection, the bodyweight of T2DM rats was significantly lighter than normal rats (*p* < 0.01, Fig. 2e). Moreover, T2DM rats required more water and feed than normal rats within 24 h (*P* < 0.001, Fig. 2f). As shown in Fig. 2g, serum NO level in the T2DM group was lower than that in the normal group (*P* < 0.01). However, serum ET-1 levels in the T2DM group were higher than that in the normal group (*P* < 0.05). Besides, lipid accumulation was observed in the skin vessels, kidneys, and myocardium of T2DM rats (Fig. 2h).

### Both topical and systemic transplantation of BM-MSCs enhanced wound healing

**Fig. 3:**
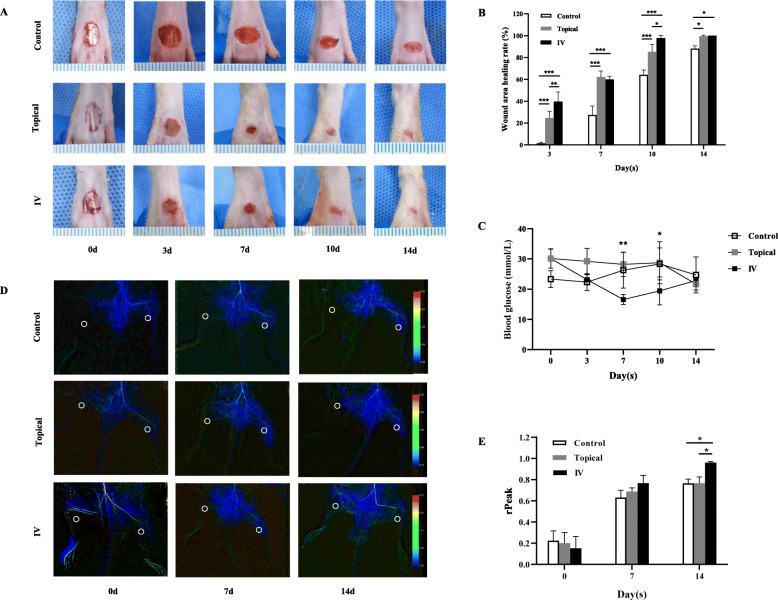
Effect on diabetic ischemic wounds in rats treated topically and systemically. Diabetic ischemic wounds (**a**), wound area healing rate (**b**), and random blood glucose levels (**c**) following PBS and BM-MSCs via topical and IV transplantation at 0, 3, 7, 10, and 14 days. The color-coded q-DSA images (**d**) and rPeak (**e**) of ROI at days 0, 7, and 14 after treatment. White circles indicate the ROI. Values are mean ± SD. *P < 0.05, **P < 0.01, ***P < 0.001

**Fig. 4:**
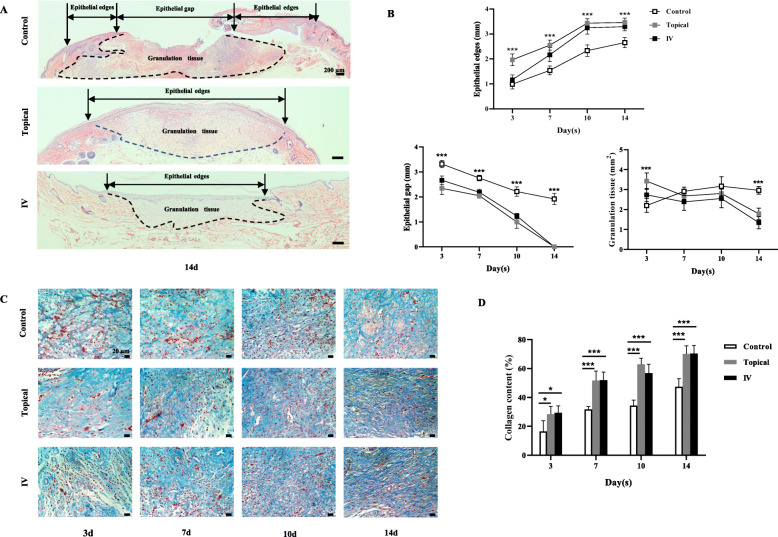
Epithelialization and granulation tissue regeneration assessment in rats treated topically and systemically. **a** H&E-stained sections of wound specimens from rats at days 14 (4 × 10). Note that the picture showed the analytical method of the length of epithelial edges, the length of the epithelial gap, and the area of granulation tissue. **b** The length of epithelial edges, the length of the epithelial gap, and the area of granulation tissue at days 3, 7, 10, and 14. **c** Collagen deposition was assessed by Masson’s trichrome (40 × 10) at days 3, 7, 10, and 14. **d** The collagen content at days 3, 7, 10, and 14. Values are mean ± SD. *P < 0.05, **P < 0.01, ***P < 0.001

As shown in Fig. 3a, b, BM-MSCs treated wounds exhibited accelerated wound closure in topical and IV groups compared with wounds in the control group at all four time points (*p* < 0.05). In addition, the healing rate in the IV group was significantly higher than that in the topical group at days 3 (*P* < 0.01) and 10 (*P* < 0.05). Histologically, both topical and IV groups wound had complete epithelialization by day 14 (Fig. 4a). As shown in Fig. 4b, the length of the epithelial edges in both treatment groups was significantly longer than that in the control group at days 7, 10, and 14 (*P* < 0.001). In contrast, the length of the epithelial gap in the control group was longer longer than that of the topical and IV groups at days 3, 7, 10, and 14 (*P* < 0.001). Meanwhile, the control group had a larger area of granulation tissue than in both topical and IV groups at day 14 (*P* < 0.001).

**Fig. 5:**
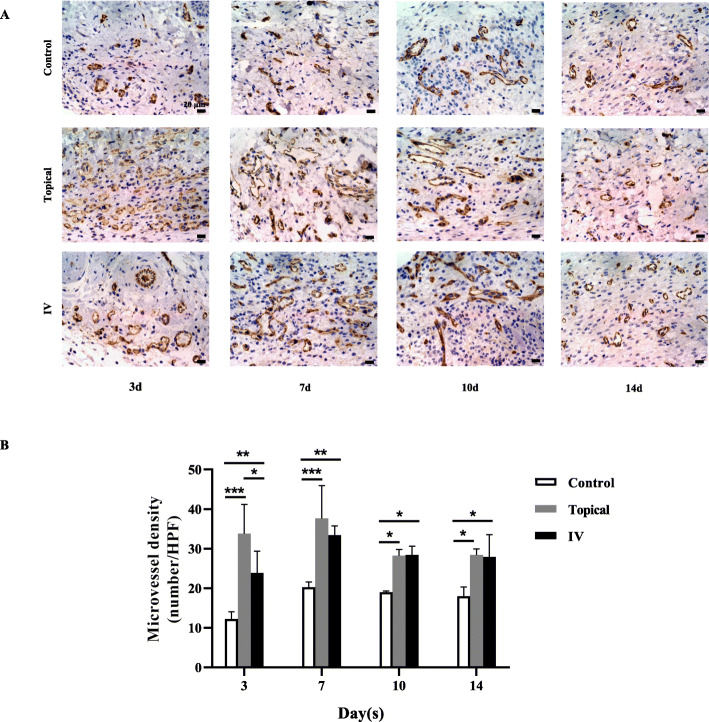
Angiogenesis assessment in the wound. **a** IHC staining of CD31 (40 × 10) showed more microvessels on the wound in the topical and IV groups compared with the control group at days 3, 7, 10, and 14. **b** The microvessel density in the wound at days 3, 7, 10, and 14. Values are mean ± SD. *P < 0.05, **P < 0.01, ***P < 0.001

Histological analysis of Masson’s trichrome staining and CD31 IHC staining sections revealed differences in the repair efficiency of the wounds among all three groups at different time points. As shown in Fig. 4a, granulation tissue in the control group was loose and accompanied by edema. By contrast, granulation tissue in topical and IV groups had tremendous collagen deposition with large wavy collagen fibers. The increase of collagen content in both topical and IV groups was greater than that of the control group at days 3 (*P* < 0.05), 7 (*P* < 0.001), 10 (*P* < 0.001), and 14 (*P* < 0.001) (Fig. 4d). Furthermore, compared with the control group, IHC staining for CD31 revealed that more angiogenic formation in the wound of both topical and IV groups at day 3 to day 14 postwounding (Fig. 5a and b).

### Systemic transplantation of BM-MSCs ameliorated hyperglycemia and improved blood perfusion of the ischemic limb

As shown in Fig. 3c, a decline of the blood glucose level was present in the IV group at days 7 and 10 but still remained the initial level at day 14. Compared with the control and topical groups, the IV group had a better blood perfusion recovery of ischemic hindlimbs at day 14 (*P* < 0.05, Fig. 3d, e).

### The homing of mKate2-labeled BM-MSCs

**Fig. 6:**
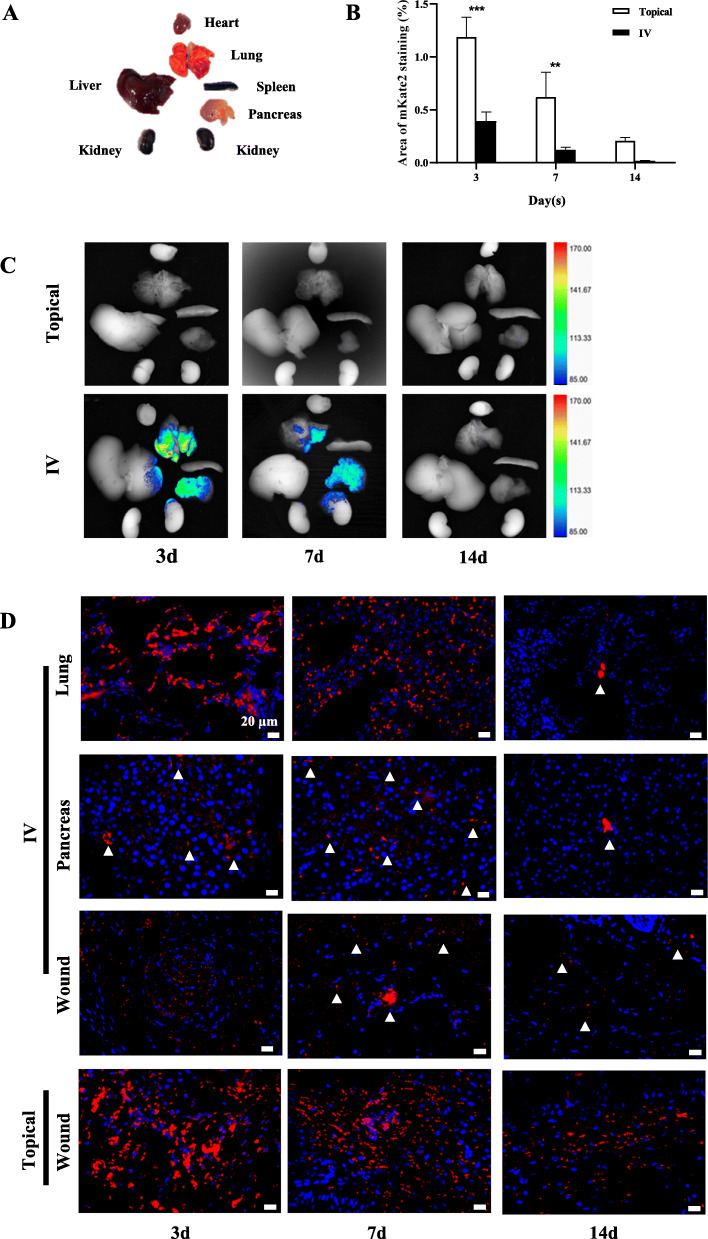
In vivo tracking of mKate2-labeled BM-MSCs during wound repair under pathological conditions. **a** The appearance of major organs harvested from model rats. **b** The area of mKate2 staining on the wound at days 3, 7, and 14. **c** Fluorescence imaging of major organs harvested at days 3, 7, and 14 after transplantation of mKate2-labeled BM-MSCs. **d** mKate2-labeled BM-MSCs distribution analysis on lung, pancreas, and wound sections at days 3, 7, and 14 posttransplantation via IV and topical, respectively (RFP IHC staining, 40 × 10). Values are mean ± SD. *P < 0.05, **P < 0.01, ***P < 0.001

Representative fluorescence imaging of major organs are shown in Fig. 6c. In the IV group, the fluorescence signals was seen in the lungs, liver, pancreas, and kidney at day 3. As time progressed, the fluorescence signals decreased and on day 14 signals were untraceable. However, no fluorescence signal could be detected in any of these organs in the topical group. As shown in Fig. 6d, mKate2 IHC analysis of the lungs, pancreas, and the wound was performed to further confirm the tracing allocation: red fluorescent protein was found in the lungs and pancreas in the IV group. Moreover, mKate2 staining was detected within the wound in both topical and IV group, with a larger stain area observed in the topical group compared to the IV group at days 3 (*P* < 0.001) and 7 (*P* < 0.01) (Fig. 6b, d).

### BM-MSCs transdifferentiated into endothelial cells and increased VEGF expression

**Fig. 7:**
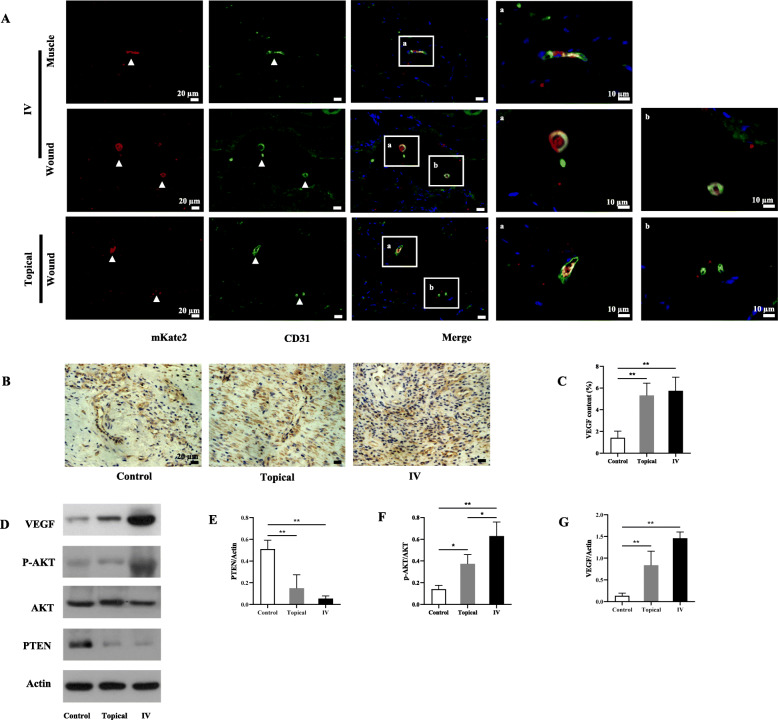
BM-MSCs enhance wound healing through transdifferentiation into endothelial cells and increasing VEGF expression. **a** mKate2 positive cells (red) were colocalized with CD31(green) in the wound site and right gastrocnemius tissues at day 14 (RFP and CD31 IHC staining, 40 × 10 or 100 × 10). **b** Representative images of VEGF in wound site at day 14 (VEGF IHC staining, 40 × 10). **c** VEGF content in the wound at day 14. **d** Western blotting analysis of VEGF, p-AKT, AKT, PTEN, and Actin expression in wound site at day 14 among three groups. The quantification of PTEN/Actin (**e**), p-AKT/AKT (**f**), and VEGF/Actin (**g**) by Western blotting. Values are mean ± SD. *P < 0.05, **P < 0.01, ***P < 0.001

The transdifferentiation capacity of MSCs and expression of VEGF were assessed in the wound at day 14. As shown in Fig. 7a, mKate2-positive cells (red) were colocalized with CD31 (green) in wound site and right gastrocnemius tissue. Furthermore, VEGF content in the control group was lower than that of the topical and IV groups (*P* < 0.01, Fig. 7b, c). Western blot analysis of the wound tissues showed a reduction of PTEN, and an increase in the amount of p-AKT and VEGF protein expression in both topical and IV groups when compared with that of the control group (Fig. 7d–g).

## Discussion

Many previous studies and clinical trials have shown that stem cells therapy and related biotechnology hold a great promise in regenerative medicine applications [25–28]. BM-MSCs, adipose-derived mesenchymal stem cells (AD-MSCs), and stromal vascular fraction cells (SVFs) have the ability to promote wound healing and soft tissue defects when used alone [29–31] or in combination with platelet rich plasma (PRP) and fat graft [32–34]. Additionally, the most recent studies reported systemically administered MSCs improve respiratory activity in patients with coronavirus disease 2019 (COVID-19) [35–37]. However, we still lack treatment guidelines given the limited evidence from comparative research, which is becoming a crucial issue hindering the clinical application of MSCs. Regarding the route of administration, topical administration was the preferred mode of administration followed by systemic administration. Topical administration is considered to be an easy and effective route for MSCs delivery to treat chronic wounds [38, 39]. Systemic administration has also indicated that MSCs could accelerate cutaneous wound healing [6] . In addition, a recent study suggests that both topical and systemic administration has the potential to facilitate wound healing under physiological conditions [17]. In this study, we sought to compare topical and systemic transplantation of BM-MSCs to investigate the proper administration method for chronic wound therapy.

We established a novel animal model of DFUs that simulates clinical symptoms. This model exhibited hyperglycemia, metabolic disorder, atherosclerosis, hindlimb ischemia, and chronic wound. The findings of the present study suggested that both topically and systemically administered BM-MSC treatment result in accelerated wound closure, facilitated reepithelialization, improved granulation, and enhanced angiogenesis. Moreover, a transient decrease in blood glucose levels was observed after systemic transplantation of BM-MSCs, as reported previously [40]. Systemic administration also improved blood perfusion in the ischemic hindlimb steadily and continuously. We believe that these systemic effects originated from the route of administration.

As the most commonly used seed cells for tissue repair and regeneration, MSCs actively respond to biological signals associated with inflammation, necrosis, and tissue injury [41]. After systemic administration, BM-MSCs were observed at the skin wound sites [42, 43], and notably, they secreted bioactive factors to recruit host repair cells [44, 45]. A plethora of chemokines, growth factors, and receptors were involved in the recruitment of MSCs [46–49]. In our study, compared with topical transplantation, BM-MSCs administered systemically was distributed in wounded tissues, such as pancreas and ischemic muscle. Our study also revealed that BM-MSCs can transdifferentiate into endothelial cells in the ischemic muscle. Therefore, that might be the reason for ameliorating hyperglycemia and improving blood perfusion of ischemic hindlimb after systemic application.

The mechanisms of MSC-based wound therapy have not been fully delineated, yet two mechanisms are generally postulated: (1) direct differentiation into skin cells [50] and (2) the secretion of trophic factors [51]. Considering the poor engraftment of MSCs at chronic wounds, it is widely believed that the therapeutic effects depend predominantly on their paracrine actions by which MSCs secrete a multitude of soluble factors, such as growth factors, immune factors, chemokines, and exosomes, to enhance the survival, recruitment, and function of wound repair cells [25]. The latest research suggests that systemic transplantation of MSCs have the potential to promote wound repair possibly by paracrine effect; however, topical transplantation of MSCs enhanced wound repair due to redistribution in the wound bed [17]. In this article, we showed that both the topical and systemic transplantation of BM-MSCs have the ability to migrate into wound sites and transdifferentiate into endothelial cells. Furthermore, compared with systemic administration, more BM-MSCs were found in the wound after topical administration. However, topical and systemic transplantation of BM-MSCs had no significant differences in wound healing. One explanation may be that systemic delivery of MSCs exerts a stronger paracrine effect than topical administration.

It is believed that angiogenesis is an important part of tissue repair [52]. As a potent growth and angiogenic cytokine, VEGF is involved in the formation and maturation of blood vessels [53]. The results from this study showed that VEGF expression increased in wounds after BM-MSC transplantation. It is established that the AKT signaling pathway is an important pathway regulating angiogenesis [54]. p-AKT activates VEGF signaling pathway, which leads to cell proliferation and angiogenesis [55]. In contrast, PTEN inhibits AKT signaling pathway through dephosphorylation [56]. In the present study, the wound following BM-MSC treatment exhibited the level of p-AKT increased while the level of PTEN decreased. Our findings demonstrate that BM-MSCs improve wound healing through regulating angiogenesis by targeting the PTEN/AKT pathway.

## Conclusion

In summary, we demonstrated that both topical and systemic transplantation of BM-MSCs accelerated wound healing remarkably under pathological conditions. This was evidenced in a diabetic ischemic wound model. At the same time, systemic administration has the potential to ameliorate hyperglycemia and repair the damaged tissue. The mechanism involved transdifferentiation into endothelial cells, stimulating VEGF secretion and activating the AKT signaling pathways. The obtained data provide new evidence for the potential application of MSC transplantation in chronic wound therapy.

## Data Availability

All data generated or analyzed during this study are included in this published article.

## Abbreviations

MSCs: Mesenchymal stem cells
T2DM: Type 2 diabetes mellitus
BM-MSCs: Bone marrow-derived mesenchymal stem cells
IV: Intravenous
IHC: Immunohistochemistry
VEGF: Vascular endothelial growth factor
DFUs: Diabetic foot ulcers
PAD: Peripheral arterial disease
STZ: Streptozotocin
DMEM: Dulbecco’s modified Eagle’s medium
FBS: Fetal bovine serum
pen/strep: Penicillin-streptomycin
CD29: Cluster of differentiation 29
CD44: Cluster of differentiation 44
CD90: Cluster of differentiation 90
CD34: Cluster of differentiation 34
CD45: Cluster of differentiation 45
IPGTTs: Intraperitoneal glucose tolerance tests
IPITTs: Intraperitoneal insulin tolerance tests
ELISA: Enzyme-linked immunosorbent assay
NO: Nitric oxide
ET-1: Serum endothelin-1
PBS: Phosphate-buffered saline
q-DSA: Quantitative digital subtraction angiography
ROI: Region of interest
H&E: Hematoxylin and eosin
HPF: High power field
CD31: Cluster of differentiation 31
DAPI: 4′,6-Diamidino-2-phenylindole
PTEN: Phosphatase and tensin homolog
p-AKT: Phosphorylation of AKT
AD-MSCs: Adipose-derived mesenchymal stem cells
SVFs: Stromal vascular fraction cells
PRP: Platelet rich plasma
